# Isobaric Tags for Relative and Absolute Quantification-Based Proteomics Reveals Candidate Proteins of Fat Deposition in Chinese Indigenous Sheep With Morphologically Different Tails

**DOI:** 10.3389/fgene.2021.710449

**Published:** 2021-11-15

**Authors:** Caiye Zhu, Heping Cheng, Na Li, Tiaoguo Liu, Youji Ma

**Affiliations:** College of Animal Science and Technology, Gansu Agricultural University, Lanzhou, China

**Keywords:** iTRAQ, fat deposit, type of tail, sheep, bioinformatics analysis

## Abstract

**Background:** Chinese indigenous sheep can be classified into two types according to their tail morphology: fat-rumped and thin-tailed sheep, of which the typical breeds are Altay sheep and Tibetan sheep, respectively.

**Methods:** To identify the differentially expressed proteins (DEPs) underlying the phenotypic differences between tail types, we used isobaric tags for relative and absolute quantification (iTRAQ) combined with multi-dimensional liquid chromatography tandem-mass spectrometry (LC-MS/MS) technology to detect candidate proteins. We then subjected these to a database search and identified the DEPs. Finally, bioinformatics technology was used to carry out Gene Ontology (GO) functional and Kyoto Encyclopedia of Genes and Genomes (KEGG) pathway analyses.

**Results:** A total of 3,248 proteins were identified, of which 44 were up-regulated and 40 were down-regulated DEPs. Analyzing their GO function terms and KEGG pathways revealed that the functions of these DEPs are mainly binding, catalytic activity, structural molecule activity, molecular function regulator, and transporter activity. Among the genes encoding the DEPs, *APOA2*, *GALK1*, *ADIPOQ*, and *NDUFS4* are associated with fat formation and metabolism.

**Conclusion:** The *APOA2*, *GALK1*, *ADIPOQ*, and *NDUFS4* genes may be involved in the deposition of fat in the tail of sheep. This study provides a scientific basis for the breeding of thin-tailed sheep.

## Introduction

To enable their adaptation to various environments, long-term natural selection has produced sheep breeds differing starkly in their individual phenotypes. The difference in tail type is one of the main changes in the evolutionary process of sheep. Studies have shown that fat-tailed sheep emerged 5,000 years ago, having gradually formed after the long-tailed Asian thin-tailed sheep were first domesticated (Moradi et al., 2012). However, fat-tailed sheep now account for about 25% of the total number of sheep in the world and are widely distributed; in China, 80% of sheep breeds are fat-tailed sheep (Wei et al., 2015). The unique way of depositing fat in the tail or buttocks of sheep is considered a key change following the domestication process (Ryder, 1991). It is generally believed the fat stored in the tail can produce enough energy in extremely harsh geographical and climatic environments, thus playing a crucial role in the survival of sheep (Farahani et al., 2010). Almost all fat-tailed sheep have better adaptability and better resistance to stress than other local breeds of sheep (Moradi et al., 2012). Fat-tailed sheep have most of the fat deposited in their tail, which invariably lessens fat deposition in other parts of the carcass, thereby affecting meat quality, because more feed is required to deposit fat than to produce meat (Negussie, 2003). It is estimated that the forage consumed to produce 1 kg of fat can yield 2 kg of lean meat (Gan et al., 2013). The fat deposited in the tail not only changes the quality of the lamb but also hinders the breeding of sheep, diminishing the economic benefits of raising them.

Isobaric tags for relative and absolute quantification (iTRAQ) are a proteome quantification technology with high throughput and high sensitivity. This technology uses a variety of isotope reagents to label the N-terminus of protein polypeptides or lysine side chain groups and simultaneously analyzes the protein expression between two and eight samples *via* high-resolution mass spectrometry; it is a high-throughput screening technique commonly used in quantitative proteomics in recent years (Lu et al., 2017; Wang et al., 2017; Yang et al., 2017). For example, Zhang et al. (2019) used iTRAQ technology to identify 30 differential proteins in chicken liver tissue under different concentrations of ammonia and Forde et al. (2014), Yang et al. (2017) used iTRAQ and liquid chromatography tandem-mass spectrometry (LC-MS/MS) technology to process protein extracts from the uterine fluid of pregnant cows at different time periods (10, 13, 16, and 19 days of pregnancy), finding significant changes in protein content of the uterine fluid across these four experimental periods. He et al. (2018) performed an iTRAQ-based quantitative proteomic analysis of horn tissues from both scurs and normal two-horned and four-horned individuals; their results indicated that the PI3K-Akt signaling pathway was the most significant, possibly affecting the formation of the extracellular matrix in horn that ultimately leads to the development of deformed horn tissue. Xu et al. (2019) used iTRAQ to identify critical proteins affecting milk fat in dairy cattle. Using the iTRAQ approach, Li et al. (2017) examined 27 fiber samples representing nine fiber types from sheep and goats and found differentially abundant proteins that are important to the structural components of hair, as well as some genes related to hair growth and fatty acid synthesis.

## Materials and Methods

### Sample Collections

**FIGURE 1:**
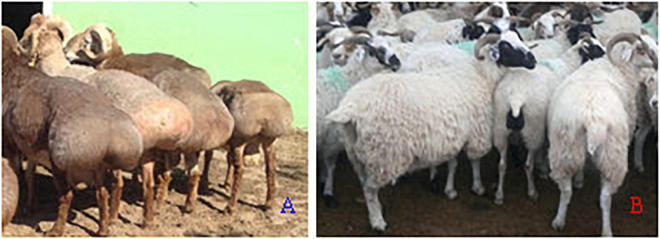
**(A)** Altay sheep with their fat-rumped tail and **(B)** Tibetan sheep with the thin-tailed type.

Three Altay sheep were randomly selected from Altay in Xinjiang Province and three Tibetan sheep from Tianzhu in Gansu Province (Figure 1). The tail length, width, and circumference of Altay sheep are 22 ± 2.7, 36 ± 1.8, and 98 ± 4.3 cm, respectively. The tail length, width, and circumference of Tibetan sheep are 15 ± 3.2, 4 ± 1.1, and 7 ± 0.3 cm, respectively. Approximately 0.5 g of tail fat tissue was removed from each sheep. After rinsing each tissue sample with normal saline, it was stored on dry ice and brought to the laboratory, where all samples were stored at −80°C until used.

### Protein Extraction and Peptide Digestion

Protein extraction was done using the SDT (4% SDS, 100 mM Tris/HCL pH 7.6, and 0.1 M DTT) cleavage method (WisNiewski et al., 2009), with the BCA method used for protein quantification. Next, an appropriate amount of protein was taken from each sample for its attempted digestion by the filter-aided sample preparation (FASP) method, after which the C_18_ cartridge was used to desalt the enzymatic peptides. After the peptides were lyophilized, 40 μl of dissolution was added to reconstitute them, and the peptide D_280 nm_ value finally measured.

### Isobaric Tags for Relative and Absolute Quantification Labeling

From each sample, 100 μg peptide was taken and the instructions of the AB SCIEX iTRAQ labeling kit followed accordingly, then the labeled peptides mixed.

### Strong Cation Exchange Chromatographic Classification

Each group of labeled peptides was mixed, with the AKTA Purifier 100 (AKTA avant25 Cytiva) used for classification. Buffer A consisted of 10 cM KH_2_PO_4_ and 25% ACN, at pH 3.0, and buffer B of 10 cM KH_2_PO_4_, 500 mM KCL, and 25% ACN, at pH 3.0. The chromatographic column was equilibrated with liquid A, a given sample loaded onto the chromatographic column by the injector, using a flow rate set to 1 ml/min. The liquid phase gradient went was follows: 0% B liquid for 25 min; linear gradient of B liquid from 0 to 10%, for 25–32 min; linear gradient of liquid B from 10 to 20%, for 32–42 min; linear gradient of liquid B from 20 to 45%, for 42–47 min, linear gradient of liquid B from 45 to 100%, for 47–52 min; and lastly, for 52–60 min, liquid B was maintained at 100%. After 60 min, the B liquid was reset to 0%. Absorbance at 214 nm was monitored during the elution process, and eluted components were collected every 1 min, freeze-dried, and desalted by using the C18 cartridge.

### Liquid Chromatography Tandem-Mass Spectrometry

Each graded sample was separated by the high-performance liquid chromatography (HPLC) liquid phase system Easy nLC by using a nanoliter flow rate. Buffer A consisted of 0.1% formic acid in water, and B was 0.1% formic acid-acetonitrile (acetonitrile: 84%). The chromatographic column was equilibrated with 95% liquid A. The sample was loaded onto the loading column by the autosampler, separated by the analytical column, and the flow rate was set to 300 nl/min. After the sample was separated by chromatography, mass spectrometry was performed by a Q-Exactive mass spectrometer. The detection method applied was “formal ion,” the scanning range of precursor ion was 300–1,800 m/z, the resolution of primary mass spectrometry was 70,000 at 200 m/z, the AGC target was 1e^6^, the maximum IT was 50 ms, and the dynamic exclusion time is 60 s. The mass-to-charge ratios of peptides and peptide fragments were collected accordingly as follows: 20 fragment spectra were collected after each full scan; the MS2-activation type was HCD; the isolation window was 2 m/z; the secondary mass spectrometry resolution was 17,500, at 200 m/z; normalized collision energy was 30 eV; and the underfill ratio was 0.1%.

### Protein Identification and Quantitative Analysis

The ensuing raw data from the mass spectrometry analysis were contained in a RAW file. Both Mascot v2.2 (Carmona et al., 2015) and Proteome Discover v1.4 software (Kim et al., 2014) were used for database checking and quantitative analysis. Each protein contained at least one specific peptide during its identification. The screening criteria for differential proteins were a *P* < 0.01 with an fold-change (FC) ≥ 2 or ≤ 0.85 (Götz et al., 2008).

### Bioinformatics Analysis

We used the Blast2GO to perform a Gene Ontology (GO) annotation of the target protein collection. This process includes sequence alignment, GO mapping, GO annotation, and InterProScan annotation augmentation. The KAAS [Kyoto Encyclopedia of Genes and Genomes (KEGG) Automatic Annotation Server] software was used to annotate the KEGG pathways of the target protein collection against the KEGG database.^1^ Fisher’s exact test was used to compare the distribution of each GO classification or KEGG pathway in the target protein collection versus the overall protein collection. GO functional terms and enriched KEGG pathways with *P* < 0.05 were considered significant.

### Protein Cluster Analysis

First, the quantitative information of the target protein collection was normalized [normalized to (−1, 1) interval]. Next, we used the “Complexheatmap” package for the R software platform (v3.4) to simultaneously categorize the two dimensions of sample and protein expression (distance algorithm: Euclidean and connection method: average linkage), from which a hierarchical clustering heat map was generated.

### Protein Interaction Network Analysis

Based on the information available in the IntAct^2^ or STRING^3^ database, the target protein or an indirect interaction relationship was searched for. Cytoscape software (v3.2.1) was used to generate an interaction network and analyze it.

### Western Blot Verification

For this assay, 50–100 mg tail fat tissue was placed in an Eppendorf tube and shredded. Then, 1 ml of the tissue cell lysate was put on ice for 3 h and then centrifuged at 12,000 rpm, at 4°C for 10 min, whose supernatant fluid was collected for use in another new EP tube. A total protein sample of about 10 μl was taken, to which 10 μl of loading buffer was added, and then boiled 3–5 min to denature the protein. Next, SDS-PAGE gel electrophoresis was conducted, and protein transferred to a PVDF membrane. Finally, according to the antigen–antibody reaction, a western blot analysis of the target protein was carried out.

## Results

### Protein Profile by the Isobaric Tags for Relative and Absolute Quantification Analysis

**FIGURE 2:**
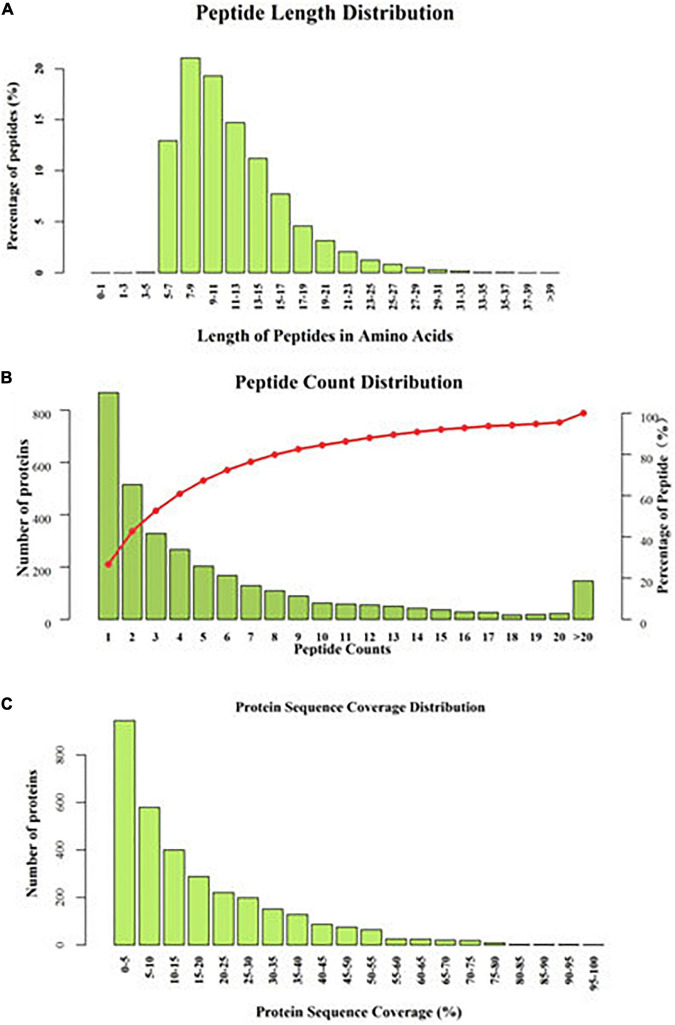
Information for the peptides and proteins identified by iTRAQ **(A–C)**.

By comparing the information obtained by iTRAQ proteomics technology and the database results, 3,248 proteins were identified from 20,120 peptides by searching against the Uniprot_Ovis_aries_27827_20180612 database. According to their segment length distribution, these peptides mainly consisted of 7–21 amino acids (Figure 2A), a reasonable length, indicating the data set is of high quality. Most protein peptides had counts of 1–13 (Figure 2B). Protein sequence coverage is shown in Figure 2C.

### Screening of Differentially Expressed Proteins

**FIGURE 3:**
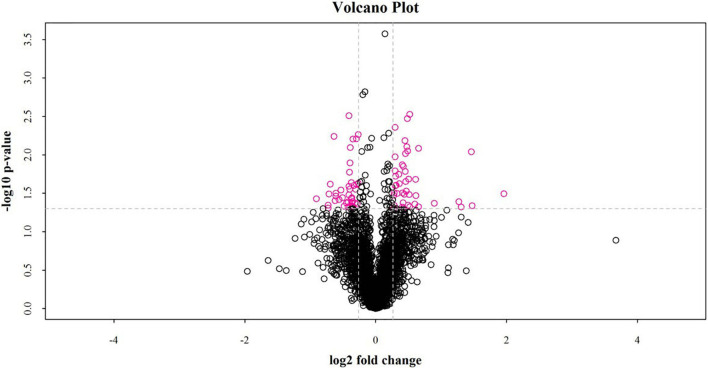
Volcano plot of differentially expressed proteins between fat-rumped and thin-tailed sheep. The red dots in the picture are proteins found to be significantly different, and the black dots are proteins with no such difference.

Between the fat-rumped and thin-tailed sheep, 44 up-regulated and 40 down-regulated proteins were identified, giving a total of 84 differentially expressed proteins (DEPs). A volcano graph (Figure 3) was drawn using two factors: the protein expression difference fold-change and the *P*-value from the *t*-test.

### Cluster Analysis of Differentially Expressed Proteins

**FIGURE 4:**
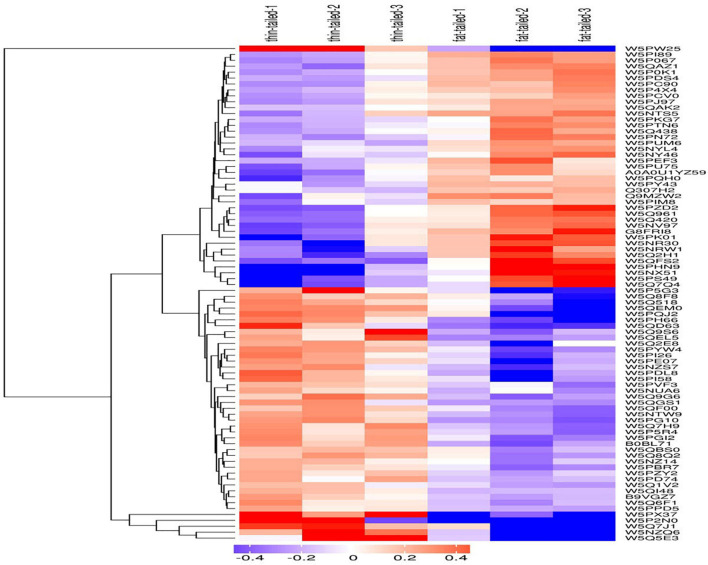
Results from the cluster analysis of differentially expressed proteins in the thin-tailed versus fat-tailed sheep. The logarithm of protein expression in different samples is displayed in different colors on the heat map. Red represents significantly up-regulated proteins, blue represents significantly down-regulated proteins, and gray represents those proteins lacking quantitative information.

In this study, the hierarchical clustering algorithm was used to perform cluster analysis on the DEPs of the comparison group. These results were displayed in the form of a heat map. As Figure 4 shows, using criteria of a fold-change greater than 1.2 times and *P* < 0.05 for the *t*-test, the DEPs obtained by the screening can effectively separate the comparison components. Accordingly, this supports the screening rationale for DEPs we employed.

Among the corresponding differentially expressed genes (DEGs) for the proteins, we found some genes related to fat synthesis and metabolism (Table 1).

**TABLE 1:** Fat-related genes found in tail tissue of sheep.

**Protein name**	**Gene name**	***P*-value**	**Functional description**
NADH:ubiquinone oxidoreductase subunit S4	*NDUFS4*	0.022	Intramuscular fat deposition
Galactokinase 1	*GALK1*	0.024	Related to fatty liver
Apolipoprotein A2	*APOA2*	0.042	Fat deposition
Adiponectin	*ADIPOQ*	0.033	Regulates fat metabolism

### Bioinformatics

### Gene Ontology Enrichment Analysis

**FIGURE 5:**
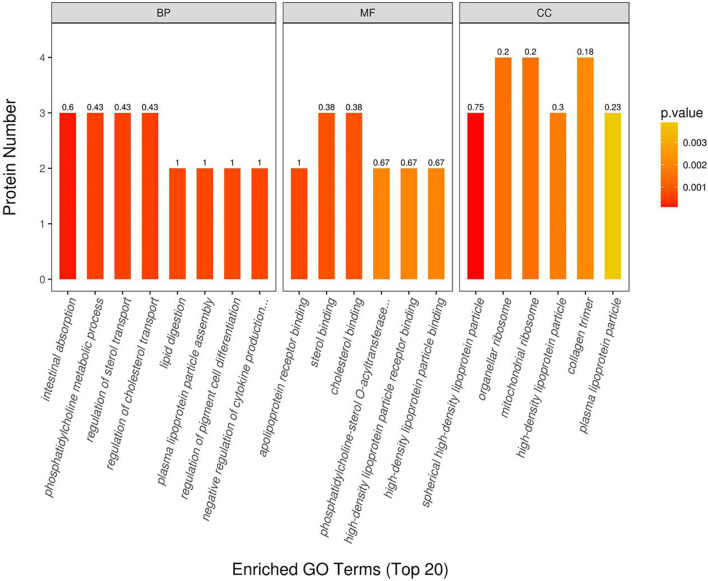
Gene Ontology (GO) annotation between thin-tailed and fat-tailed sheep. BP, biological process; MF, molecular function; CC, cell component.

We used Blast2GO^4^ software (Götz et al., 2008) to perform the GO functional annotation on all proteins identified in this study and then implemented a GO enrichment analysis of DEPs (tested by Fisher’s exact test method), as shown in Figure 5. These results uncovered important biological processes, such as intestinal absorption, regulation of sterol transport, regulation of cholesterol transporter, phosphatidylcholine metabolic process, and plasma lipoprotein particle assembly. In terms of molecular functions, those of apolipoprotein receptor binding, sterol binding, cholesterol binding, high-density lipoprotein particle binding, and high-density lipoprotein particle receptor binding were found dominant in sheep tail tissue. Localized proteins, such as spherical high-density lipoprotein particle, organellar ribosome, mitochondrial ribosome, high-density lipoprotein particle, and collagen trimer, were also all changed significantly between the thin-tailed and fat-tailed comparison group.

### Kyoto Encyclopedia of Genes and Genomes Pathway Enrichment Analysis

**FIGURE 6:**
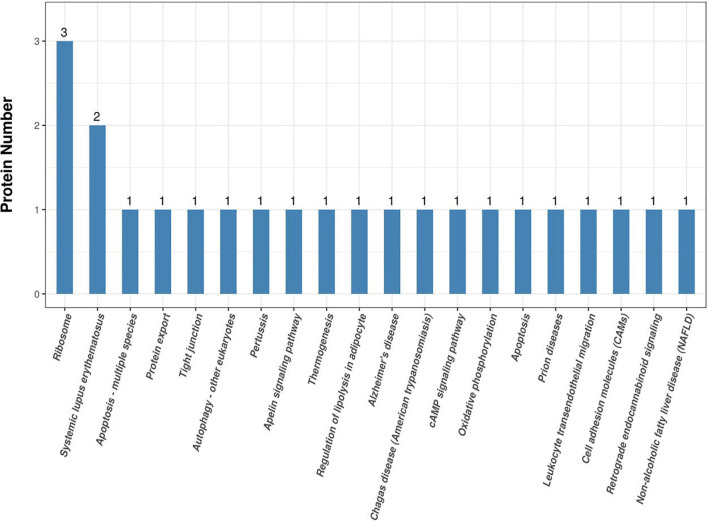
Kyoto Encyclopedia of Genes and Genomes (KEGG) pathway results.

Annotating the KEGG pathway of proteins that were significantly differentially expressed helped us to better understand the metabolic or signaling pathways that these proteins may be involved in. Specifically, it revealed the series of changes in proteins that occur from the cell surface to the nucleus. These results are shown in Figure 6. The most significant enrichment occurred in the ribosome. They are involved in regulation of transcription, DNA template, cytoplasmic translation, rRNA processing, protein serine/threonine kinase activity, viral nucleocapsid, cytosolic large ribosomal subunit, positive regulation of transcription from RNA polymerase II promoter, herpes simplex infection, viral carcinogenesis, and Ras signaling pathway.

### Differential Protein Interaction Network Analysis

**FIGURE 7:**
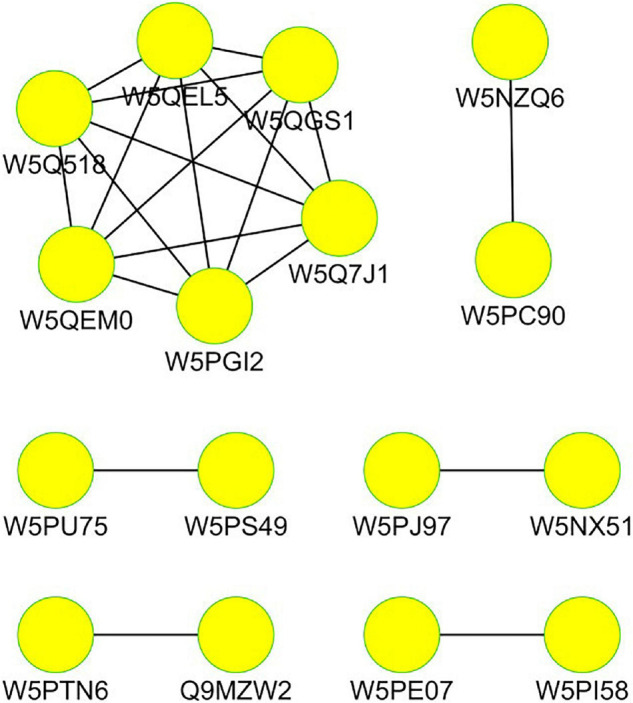
Interacting network of the differentially expressed proteins in the tails of sheep.

Cytoscape software was used to analyze the network interaction functioning of the DEPs; the results are given in Figure 7. Each functional module is composed of multiple functional nodes. In the network interaction diagram, each functional node is a differential protein, and the functional nodes mapped to important functional modules are core differential proteins.

### Western Blot Validation

**FIGURE 8:**
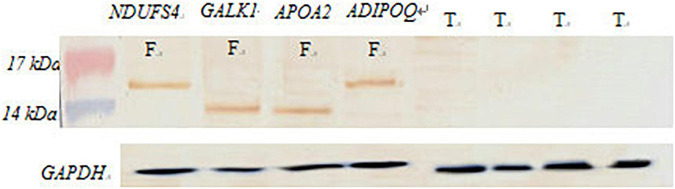
Western blot verification. F, fat-rumped sheep; T, thin-tailed sheep.

We extracted the adipose tissue protein in the tail of the fat-tailed sheep group and the thin-tailed group and detected the DEGs by western blot after an SDS-PAGE (Figure 8). The proteins were subsequently transferred onto PVDF (GE Healthcare, United Kingdom) and then blocked in 5% non-fat milk (Millipore, United States). The antibodies for WB were aggrecan mouse monoclonal antibody and rabbit polyclonal antibody (Abcam, Cambridge, United Kingdom); goat anti-rabbit IgG and goat anti-mouse IgG were labeled with horseradish peroxidase (HRP) as secondary antibodies.

These results showed that the *NDUFS4*, *GALK1*, *APOA2*, and *ADIPOQ* genes are differentially expressed in tissues between fat-rumped and thin-tailed sheep.

## Discussion

In this study, Altay and Tibetan sheep were used as experimental groups. Altay sheep have fat-rumped tails whereas the Tibetan sheep have thin tails, and the two breeds are distributed in different regions of China. Altay sheep are mainly distributed in Fuhai and Fuyun counties in the Altay region of Xinjiang Uygur Autonomous Region. The production area is dominated by typical continental climate, with an annual average temperature of 4.0°C, an extreme minimum temperature of −42.7°C, an annual snow cover of 200–250 days, and a snow thickness of 15–20 cm. The fat deposited on the buttocks forms a rounded fat hip, which is wide, straight, and rich. There is a shallow groove in the middle of the lower edge of the fat hip, which is divided into two symmetrical halves. Tibetan sheep are native to the Qinghai–Tibet Plateau and are mainly distributed in the Tibet Autonomous Region and Qinghai. The central production area is located at 26°50′–36°53′ north latitude and 78°25′–99°06′ east longitude. It is located in the southwestern part of the Qinghai–Tibet Plateau with an average elevation of over 4,000 m. The climate is characterized by long sunshine, strong radiation, low temperature, large temperature difference, clear and wet long night rain, dry winter and spring, high wind pressure, low air pressure, and low oxygen content. The two breeds have different genetic backgrounds; the Altay sheep belong to the Kazakh sheep line and the Tibetan sheep belong to the Tibetan sheep line, making genes associated with the different tail phenotypes easier to detect. Here we successfully used iTRAQ technology to analyze the differential proteins in tail fat tissue samples of these breeds differing in tail types. These proteins are not comprehensive using the method. The enrichment analysis only uses these detectable proteins. Therefore, the enrichment analysis is not accurate.

The protein interaction network is constituted by the interaction of proteins to participate in all aspects of life processes such as biological signal transmission, gene expression regulation, energy and material metabolism, and cell cycle regulation. A systematic analysis of the interactions of a large number of proteins in biological systems is of great significance for understanding the working principles of proteins in biological systems, understanding the reaction mechanism of energy material metabolism, and understanding the functional connections between proteins. In mammals, fatty acid metabolism is intricate, and some key proteins play a vital role (Ivessa et al., 2013). In the analysis of differential protein pathways, it is significantly enriched in pathways such as metabolism and fat synthesis pathways, and these differential proteins regulate different biological pathways and ultimately lead to the difference in fat deposition in the tails of fat-tailed sheep and lean-tailed sheep. Apolipoprotein A2 (*APOA2*) plays an important role in fat metabolism. In recent years, the *APOA2* gene has been used often as a candidate gene for fat deposition, and its genetic polymorphism distribution among different poultry was studied to find genetic markers related to production traits to inform and guide breeding production. A research by Song et al. (2013). demonstrated that the *APOA2* gene is a also a key gene for fat deposition in pigs. This gene was also identified in our study, so it could also be involved in fat deposition that occurs in the tail of sheep. The *NDUFS4* gene encodes the respiratory chain NADH dehydrogenase in the mitochondrial membrane, and previous studies have shown that the mitochondria play a key role in adipocyte differentiation. Mckay et al. (2003) found that chemical inhibition of the mitochondrial respiratory chain leads to reduced fat deposition in nematodes and 3T3-L1 cell lines. A work by Zhang et al. (2019) proved that the transcript abundance of *NDUFS4* mRNA expression in the longest back (posterior) muscle was significantly higher in the high-intramuscular fat (IMF) than low-IMF Laiwu pig, and its expression in the IMF-rich Laiwu pig also significantly exceeded that of the IMF-poor large white pig, prompting the authors to speculate that *NDUFS4* affects the deposition of IMF (Song et al., 2013). Adiponectin (*ADIPOQ*) is the first adipose tissue found in mice, by Scherer et al. (1995) who named it as a fat cell complement-related protein. *ADIPOQ* plays an important role in regulating fat metabolism, mainly by binding to receptors and acting on target tissues to exert biological effects. Animal breeds, in having different growth periods and tissue parts, as well as physiological conditions, are expected to influence how *ADIPOQ* regulates lipid metabolism, which in turn affects fat deposition in animals.

## Conclusion

The iTRAQ technology was used to compare the fat proteins in the tail of Altay sheep and Tibetan sheep, and the DEPs obtained were analyzed. It is speculated that certain genes, namely, *APOA2*, *GALK1*, *ADIPOQ*, and *NDUFS4*, could be involved in the regulation of fat deposition in the tail of sheep.

## Data Availability Statement

The mass spectrometry proteomics data have been deposited to the ProteomeXchange Consortium via the PRIDE (Carmona et al., 2015) partner repository and data are available via ProteomeXchange with identifier PXD029488.

## Ethics Statement

The animal study was reviewed and approved by the Gansu Agricultural University (Lanzhou, China), approval no. GSAU-AEW-2017-0003.

## Author Contributions

CZ conceived and designed the experiments, performed the experiments, and wrote the manuscript. YM, NL, TL, and HC analyzed the data and contributed reagents, materials, and analysis tools. All authors contributed to the article and approved the submitted version.

## Conflict of Interest

The authors declare that the research was conducted in the absence of any commercial or financial relationships that could be construed as a potential conflict of interest.

## Publisher’s Note

All claims expressed in this article are solely those of the authors and do not necessarily represent those of their affiliated organizations, or those of the publisher, the editors and the reviewers. Any product that may be evaluated in this article, or claim that may be made by its manufacturer, is not guaranteed or endorsed by the publisher.

## Footnotes

1
http://www.genome.jp

2
http://www.ebi.ac.uk./intact/main.xhtml

3
http://string-db.org/

4
https://www.blast2go.com/
